# The effect of laser pulse evolution on down-ramp injection in laser wakefield accelerators

**DOI:** 10.1038/s41598-024-69049-4

**Published:** 2024-08-19

**Authors:** Arohi Jain, Samuel R. Yoffe, Bernhard Ersfeld, George K. Holt, Devki Nandan Gupta, Dino A. Jaroszynski

**Affiliations:** 1 Department of Physics and Astrophysics, University of Delhi, Delhi, 110 007 India; 2 Department of Physics, SUPA and University of Strathclyde, Glasgow, G4 0NG UK

**Keywords:** Physics, Plasma physics, Plasma-based accelerators

## Abstract

Electron self-injection in laser wakefield accelerators (LWFAs) is an important determinator of electron beam parameters. Controllable and adjustable LWFA beams are essential for applications. Controlled injection by capturing sheath electrons can be achieved using plasma density down-ramps or bumps, which perturb the LWFA bubble phase velocity by varying the plasma frequency and by affecting relativistic self-focussing of the laser. We report on a comprehensive study, using particle-in-cell simulations, of the effect of laser pulse evolution on injection on density perturbations. We show how the LWFA can be optimised to make it suitable for use in a wide range of applications, in particular those requiring short duration, low slice-emittance and low energy spread, and high-charge electron bunches.

## Introduction

The rapid development of laser wakefield accelerators (LWFAs)^1^ is attracting the interest of research communities because of their potential as compact tools for scientific discovery and applications in energy, healthcare, manufacturing etc. High charge, high-energy electron bunches^2^ are used in radiobiology, radiotherapy, pharmaceutics, chemistry, and industrial radiography^3–6^. High-quality, high-energy electron beams from LWFAs^2,7–12^ are potential next-generation compact light sources^13^, such as ultra-compact X-ray free-electron lasers (FELs) and brilliant betatron sources^14–18^, which have many applications including X-ray imaging^4,19^, material structure analysis, diagnostics in high energy density physics^20,21^, photolithography and tumour treatment^22^. Electron beams can also be used to produce high photon energy bremsstrahlung radiation for high contrast industrial and medical imaging applications^23^. Most of these applications require stable, reproducible, high-quality, and energy tunable electron beams with a range of different characteristics.

The accelerating fields of a LWFA are produced by the ponderomotive force of an ultrashort intense laser pulse, which displaces plasma electrons and excites a near-luminal plasma wave with an associated wakefield that has a characteristic length equal to the plasma wavelength $\lambda _p=2\pi c/\omega _p$, where $\omega _p= \sqrt{n_e e^2/( m_e \varepsilon _0)}$ is the plasma frequency and *e*, $m_e$, and $n_e$ the electron charge, mass, and density, respectively. Particles are accelerated to high energies in millimetres to centimetres^1,24^. In the nonlinear LWFA “bubble” regime, the intense driver laser causes near-total cavitation of plasma electrons in the trailing wake, which results in fields exceed several hundreds of GV/m^25^. Self-injection of plasma electrons into this field can occur when the laser pulse power exceeds the critical power for relativistic self-focusing^26^, $P > 17.4 (\lambda _p/\lambda )^2$ GW^27^, where $\lambda$ is the laser wavelength. Self-phase modulation leads to self-steepening^28^, self-compression, which results in variations of the group velocity of the laser pulse^29^, and deformation and elongation of the bubble. This directly affects electron self-injection and beam quality; mismatch of the laser pulse in its self-guiding structure can cause oscillations of its spot size, resulting in modifications to both the bubble size and its phase velocity.

Numerous methods for self-injection of electrons into the laser wakefield have been investigated for producing low-emittance, high-brightness electron beams, mainly based on analysis of particle-in-cell (PIC) simulations^30–32^. A promising technique is ionization induced injection, where inner-shell electrons are ionized near the peak of the laser pulse at a potential that results in injection and trapping inside the wakefield^33–39^. Another interesting injection method is colliding pulse injection, where the ponderomotive force of colliding pulses boosts the electron velocity to the bubble velocity^40^, which results in energy tuneable electron beams, but this is experimentally challenging as it requires alignment and synchronization of laser pulses. However, a very simple injection method, proposed by Bulanov et al.^41^, takes advantage of a brief reduction of the bubble velocity while the laser propagates through a downward density ramp^30^. For longer density transitions (length of the down-ramp), $L_{tr} > \lambda _p$, the local phase velocity $v_p$ of the plasma wave in a one-dimensional model can be expressed as^42–44^

1 $$\begin{aligned} v_p= v_g\left( 1+\frac{\zeta }{2 n_e}\frac{dn_e}{dz}\right) ^{-1}, \end{aligned}$$

where $v_g$ is the laser group velocity, $\zeta =z-v_g t$ is the spatial coordinate in the frame co-moving with the laser pulse and $n_e(z)$ is the plasma density. The phase velocity of the plasma wave behind the laser pulse is reduced briefly in a downward density gradient ($dn_e/dz<0$ and $\zeta <0$ behind the laser pulse), which enables efficient and controllable trapping of electrons. Electrons displaced at an earlier time oscillate at a higher plasma frequency (because of the higher density) and cross the back of the bubble at a time when the plasma frequency has decreased, thus appearing in the accelerating structure. As a result, a significant fraction of electrons can be trapped in the wakefield by the end of the density transition. For sharp density transitions, where $L_{tr}< \lambda _p$ and the plasma density reduces from $n_1$ to $n_2$, the change in the plasma wavelength by the end of a density transition is given by $\Delta \lambda _p = \lambda _p [1-(n_1/n_2)^{1/2}]$^45^. Injected electrons are located at a similar phase of the wake, have similar initial energy, and are exposed to the same accelerating field, which results in a quasi mono-energetic beam. The required density profile can be created by a razor blade that partially intercepts a supersonic gas jet to create a narrow shock front with a sharp drop in density ($< \lambda _p$)^46–48^ or multiple intercepting gas jets^49^. The down-ramp injection technique for beam-driven wakefield accelerators^50–54^ and the LWFA^55,56^ has been extensively studied for generating high-quality electron beams with attosecond duration electron bunches^57^.

The properties of injected electron bunches depend on the evolution of the laser pulse in the plasma^48,57,58^. Recent experiments and PIC simulations suggest that this is due to non-linear lengthening of the plasma wave^59^, which depends on both the peak laser intensity and its waist, and to variations of the wake phase velocity, which are a function of the laser vector potential $a_0=eE_L/(m_e\omega c)$^57,60^, where $E_L$ is the electric field amplitude and $\omega$ the laser angular frequency. In addition, the front part of the laser pulse loses energy to the wakefield as it propagates due to photon deceleration^61^ and diffraction, which results in laser etching and reduction of its group velocity^62^. It is therefore expected that changes in the wake phase velocity at the density down-ramp will be influenced by the laser amplitude. As $a_0$ evolves the increased ponderomotive force will result in displacement of more charge, when forming the wake, and more momentum will be transferred to electrons that form the sheath. Both the wakefield bubble size^57,63^ and the sheath electron trajectories^64^ strongly depend on the laser pulse intensity. Thus, a larger number of electrons will be injected for higher $a_0$. We will show that variations in the laser pulse over the down-ramp strongly affect the injected charge and its phase-space distribution. The precise position of the down-ramp region becomes an important and readily adjustable parameter that can be used to optimise the electron bunch properties because of the spatio-temporal evolution of the laser pulse.

**Figure 1:**
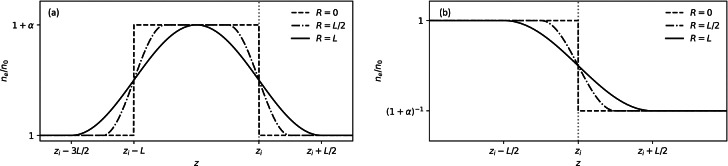
Density shapes. (**a**) Schematic showing a density bump of relative height $\alpha$ and length *L*, with the down-ramp region aligned at $z_i$ (indicated by the vertical dotted line). As the ramp length, $0 \le R \le L$, is increased and the bump profile is smoothed, while maintaining a constant full width at half height, which is aligned with position $z_i$. (**b**) A single down-ramp positioned at $z_i$, and density $n_0$ drops to $n_0/(1+\alpha )$, with the density midpoint aligned with $z_i$.

Here we use PIC numerical simulations to investigate the role of laser pulse evolution and plasma density profile on injection, for both density bumps and single down-ramps, as shown in Fig. 1. The properties of accelerated electron bunches are analysed as a function of the position, length and peak density of the down-ramp region, to establish the optimal conditions for producing high-quality beams, and more generally for controlling electron bunch properties, such as energy, charge, and total and slice emittance and energy spread. Both average (integrated) and slice values of parameters are calculated because they give a more comprehensive insight into the intrinsic quality of the self-injected electron bunches. This provides a reliable and simple method of optimising electron beam parameters for applications. We show that the minimum slice energy spread of 0.5% can be achieved for peak current of 5–6 kA and normalised slice emittance as small as $0.05\,\pi$ mm mrad.

## Results

Particle-in-cell (PIC) simulations have been performed (see “Methods”) to evaluate the influence of selected plasma density profiles on injection. We first consider a density bump with a relative peak height, $\alpha$, above the background density, and bump length *L*, as illustrated in Fig. 1a. The profile is chosen to have sinusoidal up- and down-ramps of length $0 \le R \le L$, where $R = 0$ defines a top-hat, flat-top, bump (dashed) and $R = L$ defines a fully-smoothed bump with no plateau (solid). The injected bunch characteristics are explored by varying the bump position, height, length and ramp length. To ensures the same initial laser evolution for all relative density drops, $\alpha$, we also investigate a single density down-ramp, as illustrated in Fig. 1b, where the density drops from a plateau at $n_0$ to a plateau at $n_0/(1+\alpha )$. However, the lower density plateau after the transition has an impact on subsequent acceleration due to lower accelerating fields and higher bubble phase velocities for the three different values of ramp lengths, *L*.

**Figure 2:**
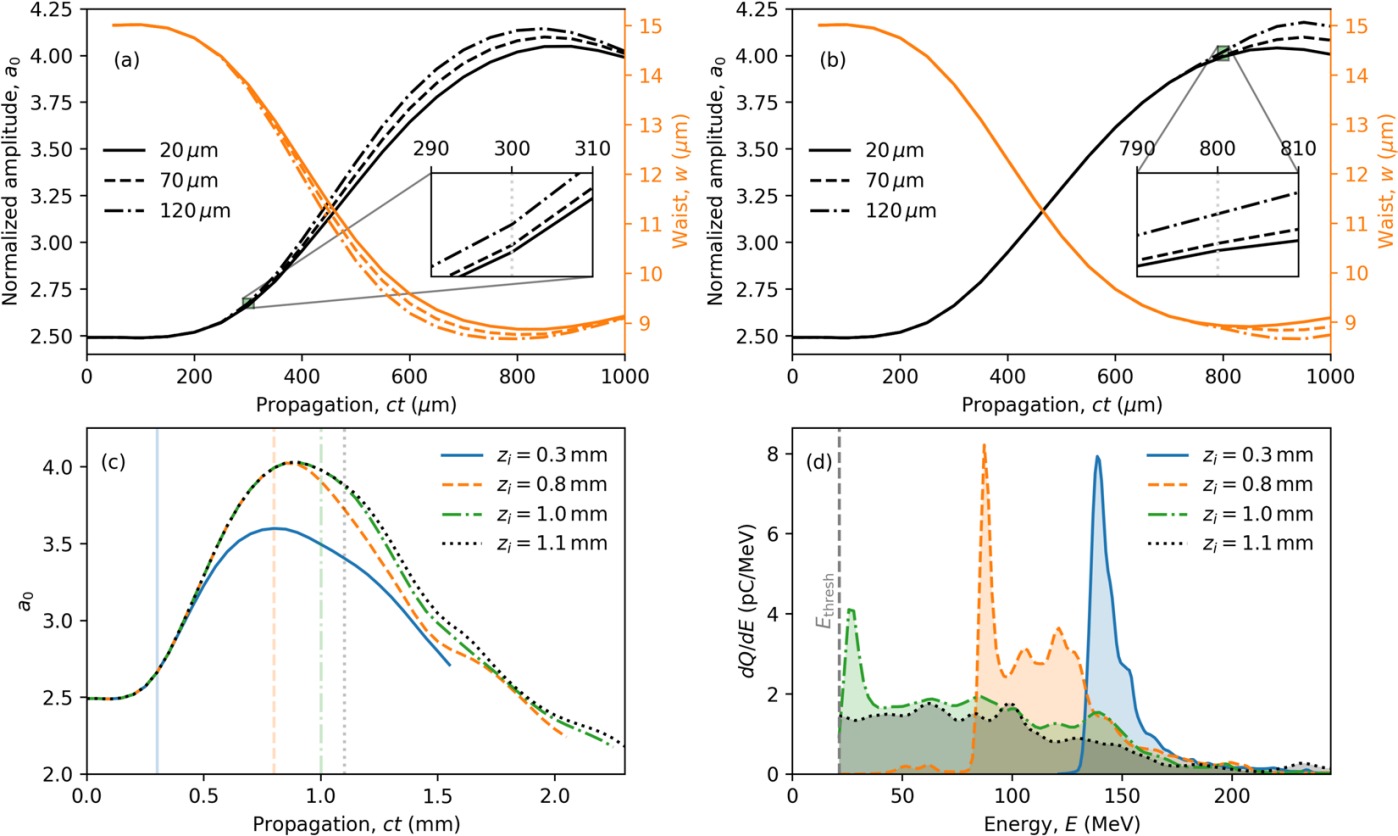
Laser pulse evolution dependence on bump length and ramp position. (**a**) and (**b**) illustrate the evolution of the laser pulse for bumps with $L = 20,70,120$ $\upmu \hbox {m}$ (solid, dashed, dot-dashed, respectively). Self-focussing of the laser pulse is enhanced with increase in bump length. The inset shows the down-ramp region magnified for (**a**) $z_i = 300$ $\upmu \hbox {m}$ and (**b**) $z_i = 800$ $\upmu \hbox {m}$. (**c**) illustrates the evolution of the laser pulse amplitude, $a_0$, for a single ($\alpha = 0.3$) down-ramp with $R = 0$ located at position $z_i$ (vertical lines). The laser pulse self-focuses in the initial higher-density region, resulting in a larger $a_0$ for later injection positions. (**d**) Electron energy spectra 1 mm after injection for various ramp positions, $z_i$.

Figure 2a,b shows the evolution of $a_0$ and the laser waist size for bump positions $z_i = 300$ $\upmu \hbox {m}$ and $z_i = 800$ $\upmu \hbox {m}$, respectively. The evolution of the laser pulse is identical up to the position where it encounters the bump. It continues to be self-focussed as it propagates through the higher density plasma bump resulting in a slight increase in the laser amplitude causing more charge to be displaced, which leads to an increase in the injected charge. Electrons also gain additional momentum from the increased ponderomotive force due to the higher intensity laser pulse, which contributes to an increase in injected charge^60^.

The evolution of the laser pulse results in a dependence of the electron bunch properties on the injection position, which is illustrated in Fig. 2c for single down-ramps (with $R = 0$ and $\alpha = 0.3$) positioned at different $z_i$. The evolution of laser pulses is identical until the point of injection, after which the drop in plasma density alters the subsequent evolution of the laser pulse because of relativistic self-focusing. Without a down-ramp, the laser pulse self-focusses to $a_0 \simeq 4$ at around $z = 0.8$ mm. Self focussing is reduced for an earlier density drop position, $z_i$, as the laser pulse propagates mainly in the lower density plasma and therefore reaches a lower maximum intensity at a slightly earlier time. In contrast, injection for later $z_i$ increases the injected charge because of the increase in $a_0$ at the time of injection (indicated by the vertical lines). Electron bunches injected near to or beyond the laser focal position experience a contraction of the bubble due to the decreasing laser intensity, and therefore the back of the bubble can catch up with and partially overtake the electron bunch^62^. When $a_0$ is large the plasma bubble size increase^65^ results in deceleration of the rear of the bunch and acceleration of its front, increasing the overall energy spread and emittance. Figure 2d shows the energy spectra 1 mm after injection, for four different injection positions, where we only record energies above $E_{\textrm{thresh}} = 20$ MeV (vertical dashed line). The properties of injected electrons depend indirectly on $a_0$ through positioning of the down-ramp. In the following sections, we discuss the electron bunch properties for both density bump and single down-ramp cases.

### Bump injection

A density modulation or bump can be used to precipitate injection by changing the phase velocity of the back of the bubble^57^. An up-ramp causes the plasma wavelength to decrease and the bubble to contract, which increases the velocity of its back thus suppressing injection. In contrast, on a down-ramp the bubble expands, which decreases its velocity. The density gradient (height and ramp length) determines the bubble velocity, see Eq. (1), which is confirmed by simulation data.

**Figure 3:**
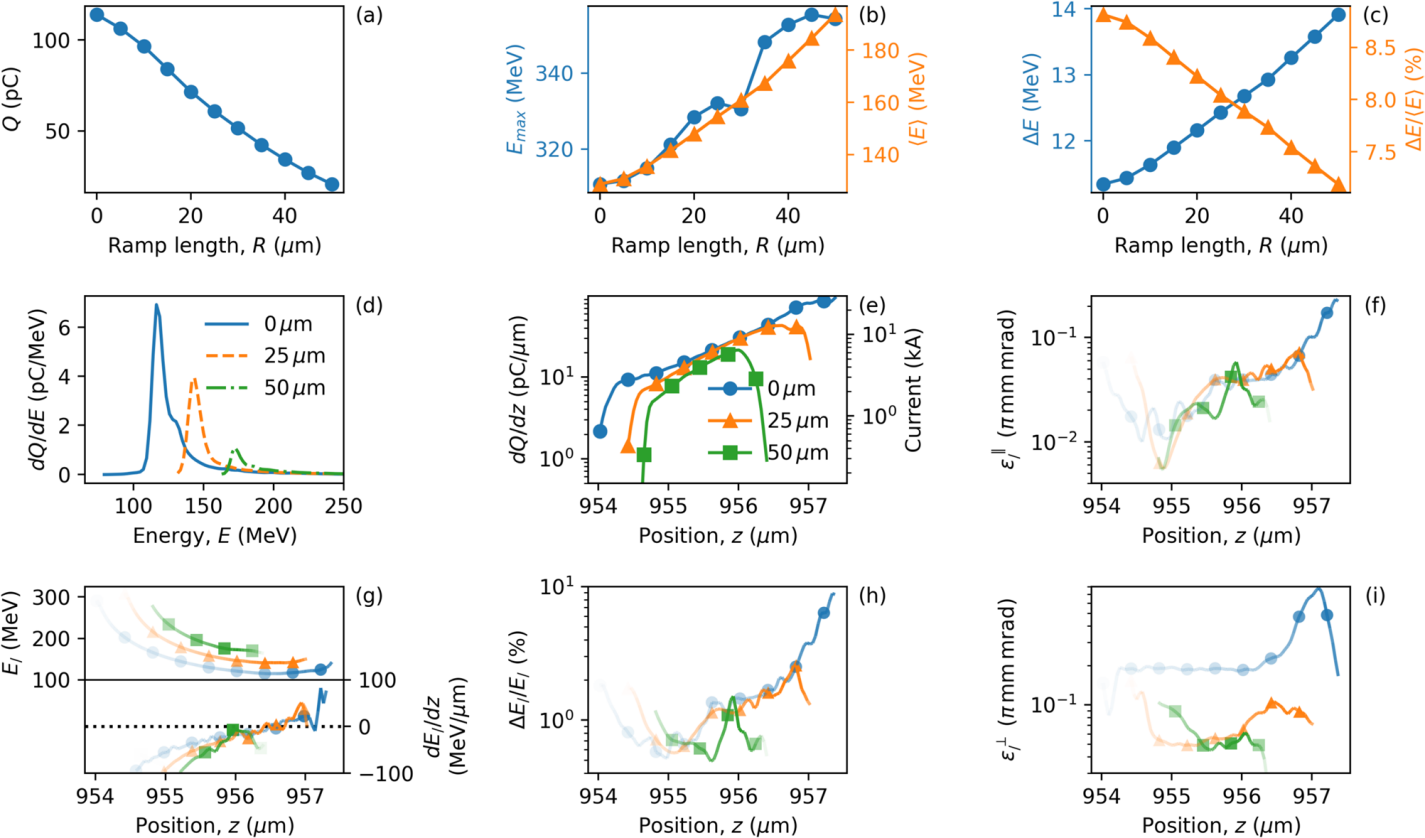
Effect of ramp length on bunch properties. Electron bunch properties for injection at $z_i = 300$ $\upmu \hbox {m}$, while varying the ramp length, *R*. (**a**–**c**) show the variation of the injected charge, energy, and energy spread, respectively. Data points indicated by circles correspond to the left axis, and triangles to the right axis. Panels (**d**,**e**) show the energy spectra and charge distribution, respectively, for three ramp lengths, $R = 0, 25, 50$ $\upmu \hbox {m}$. Properties in narrow transverse slices of electrons are presented in panels (**f**–**i**), where opacity indicates charge and the same legend as in panel (**e**) is used to identify lines. Properties are calculated at $ct = 1$ mm (700 $\upmu \hbox {m}$ after injection).

To investigate how the bunch characteristics depend on the density bump parameters, we have varied the ramp length (*R*), bump length, height and position of the density bump. We begin by varying *R*, in Fig. 3, which presents (a) the injected charge, (b) the maximum energy ($E_{max}$) and average energy ($\langle E \rangle$), and (c) the r.m.s. energy spread ($\Delta E$) and relative energy spread ($\Delta E/\langle E \rangle$). These data are calculated 700 $\upmu \hbox {m}$ after injection for a density bump of relative height $\alpha = 0.3$ at position $z_i = 300$ $\upmu \hbox {m}$. The results show that all three quantities (charge, energy, and energy spread) can be tuned almost linearly with ramp length, *R*. Within the ramp length range explored, the injected charge reduces significantly from $R=0$ to $R=50$ $\upmu \hbox {m}$, as the change in the plasma wavelength does not cause the velocity of the back of the bubble ($v_p$) to reduce significantly (refer to Eq. (1)) so that fewer electrons meet the requirements for injection. As a result, injected bunches have a lower current for $R=25$ and 50 $\upmu \hbox {m}$, as shown in Fig. 3e.

**Table 1:** Bunch parameters for different ramp lengths from Fig. 3.

Ramp length, *R* [$\upmu$m]	Figure 3h pos. of min. $\Delta E_//\langle E_/ \rangle$ [$\upmu$m]	Figure 3h $\Delta E_//\langle E_/ \rangle$ [%]	Figure 3g *E* [MeV]	Figure 3g *dE*/*dz* [MeV/$\upmu$m]	Figure 3e *dQ*/*dz* [pC/$\upmu$m]	Figure 3e Current [kA]	Figure 3f $\varepsilon _/^\parallel$ [$\pi$ mm mrad]	Figure 3i $\varepsilon _/^\perp$ [$\pi$ mm mrad]
0	955.0	0.52	156	− 50.7	12.68	3.8	0.011	0.19
25	955.1	0.57	188	− 80.4	11.32	3.4	0.012	0.05
50	955.6	0.49	187	− 45.3	15.58	4.7	0.017	0.05

Slice values, $\Delta E_//\langle E_/ \rangle$, $\varepsilon _/^\parallel$ and $\varepsilon _/^\perp$, correspond to position where $\Delta E_//\langle E_/ \rangle$ is smallest.

The bunch lengths for different ramp lengths *R* in Fig. 3e are: $\sigma _{FWHM}= 0.9$ $\upmu \hbox {m}$, $\sigma _{rms}= 2.3$ $\upmu \hbox {m}$ ($R= 0$ $\upmu \hbox {m}$); $\sigma _{FWHM}= 1.2$ $\upmu \hbox {m}$, $\sigma _{rms}= 2.2$ $\upmu \hbox {m}$ ($R= 25$ $\upmu \hbox {m}$); $\sigma _{FWHM}= 0.9$ $\upmu \hbox {m}$, $\sigma _{rms}= 1.5$ $\upmu \hbox {m}$ ($R= 50$ $\upmu \hbox {m}$). Here, $\sigma _{FWHM}$ and $\sigma _{rms}$ represent full-width half maximum and root mean square width, respectively. High charge bunches strongly deform the accelerating field through beam loading^66,67^ and their average energy decreases as the charge increases. This is clearly observed when comparing the cases $R=0, 25, 50$ $\upmu \hbox {m}$ in Fig. 3d. Other important properties calculated for electrons located within thin transverse slices, such as the slice longitudinal emittance ($\varepsilon _/^\parallel$), slice average energy ($\langle E_/ \rangle$), relative slice energy spread ($\Delta E_//\langle E_/ \rangle$), and slice transverse emittance ($\varepsilon _/^\perp$), are shown in Fig. 3f–i and their values for the lowest slice energy spread are shown in Table 1.

The energy spread is higher around the high current region of the electron bunch, and the average slice energy spread for $R = 0,\ 25$, and 50 $\upmu \hbox {m}$ is $\Delta E_// \langle E_/ \rangle = 3.09\%$, 1.42%, and 0.81%, where $\langle E_/ \rangle = 128.7, 154.5$, and 193.5 MeV, respectively. The reduced density gradient due to increasing *R* suppresses injection of electrons with lower longitudinal velocities caused by large transverse momenta. Therefore, the transverse slice emittance is lower for longer ramp lengths. In contrast, to maximize the charge in the electron bunch, a square bump with sharp density transition ($R=0$) should be chosen, at the expense of emittance. From Table 1 it can be seen that segments of the electron bunch have excellent slice parameters, e.g. slice energy spread of $\Delta E_//\langle E_/ \rangle \approx 0.5\%$, peak current of $\approx$ 4 kA and transverse slice emittance of $\varepsilon _/^\perp \approx 0.05 \,\pi$ mm mrad.

**Figure 4:**
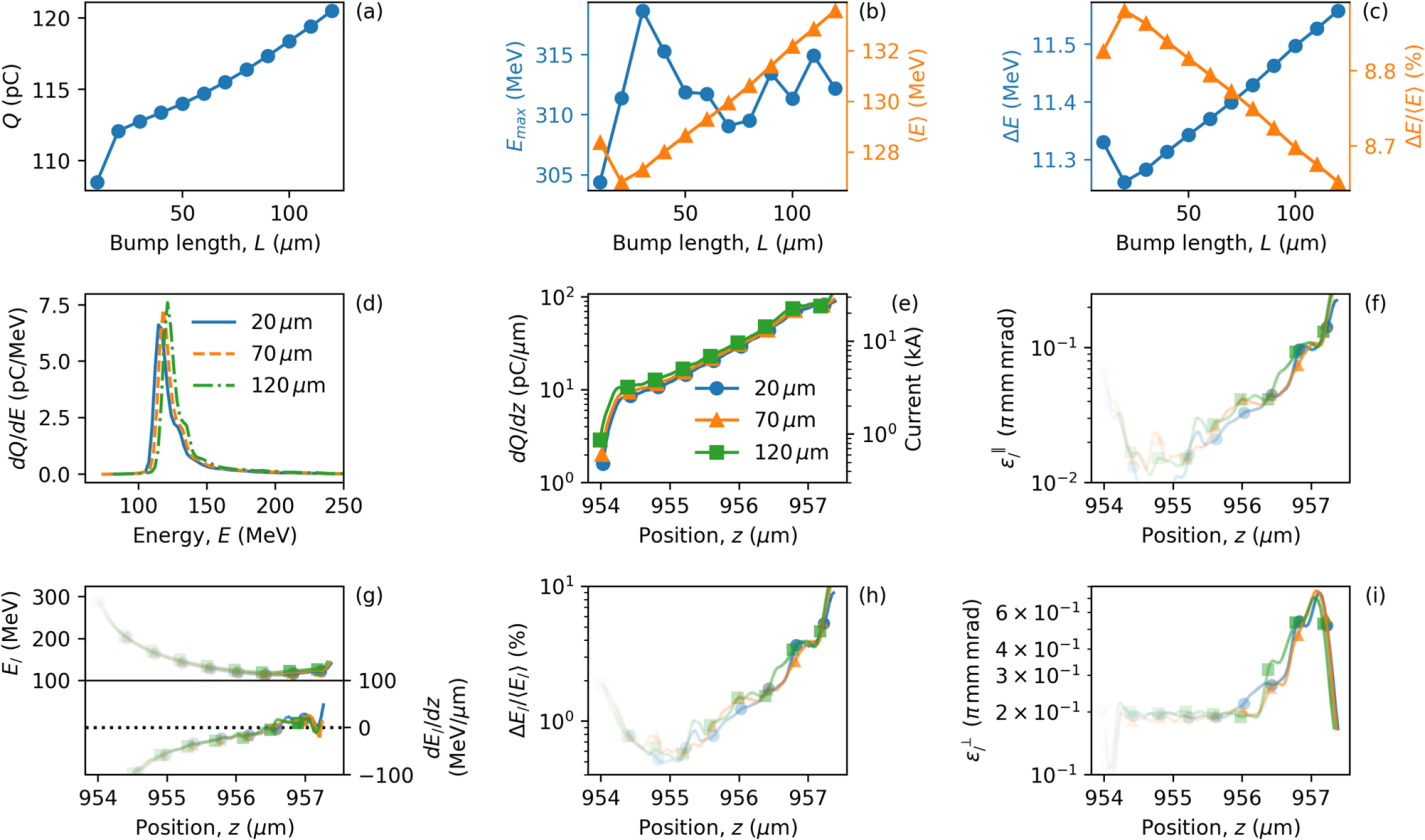
Effect of bump length on bunch properties. Electron bunch properties for injection at $z_i = 300$ $\upmu \hbox {m}$ with varying bump length and fixed bump height, $\alpha =0.3$. Panels (**a**–**c**) show the variation of the injected charge, energy, and energy spread, respectively. Data points indicated by circles correspond to the left axis, and triangles to the right axis. Panels (**d**) and (**e**) show the energy spectra and charge distribution, respectively, for three example bump lengths, $L = 20,70,120$ $\upmu \hbox {m}$. Properties of electrons contained in narrow transverse slices are presented in panels (**f**–**i**), where opacity is used to indicate charge and the same legend as panel (**e**) is used to identify lines. Properties are calculated at $ct = 1$ mm (700 $\upmu \hbox {m}$ after injection).

**Figure 5:**
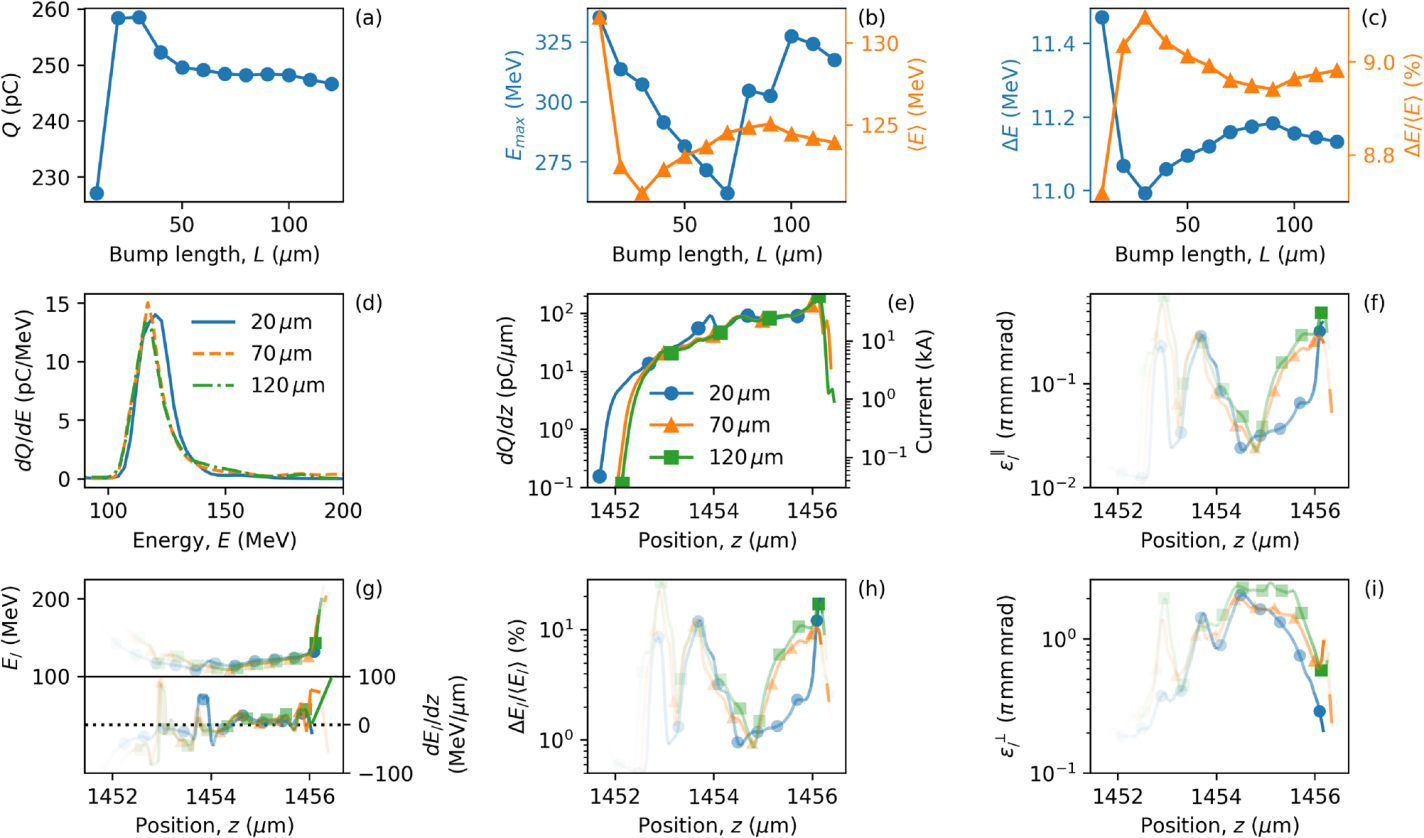
Effect of injection point on bunch properties. As Fig. 4 but for electrons injected at $z_i = 800$ $\upmu \hbox {m}$ and properties calculated at $ct = 1.5$ mm (700 $\upmu \hbox {m}$ after injection).

Figures 4 and 5 show the electron bunch properties calculated 700 $\upmu \hbox {m}$ after injection at $z_i = 300$ $\upmu \hbox {m}$ and $z_i = 800$ $\upmu \hbox {m}$, respectively. The latter corresponds to injection near the peak laser amplitude, after which the laser intensity drops. We see that the position $z_i$ has a clear impact on the injected charge – the increased laser amplitude at the point of injection leads to more charge. For $z_i = 300$ $\upmu \hbox {m}$ (Fig. 4) increasing the bump length corresponds to an increase in $a_0$ and an increase in injected charge. In contrast, for $z_i = 800$ $\upmu \hbox {m}$ (Fig. 5) increasing the bump length ($L>20$ $\upmu \hbox {m}$) causes a decrease in injected charge. In comparison, Figs. 4b and 5b also show how injection further along the laser evolution produces lower average electron energy and larger emittance, shown in Figs. 4f–i and 5f–i. Note that higher average energies are usually obtained for lower charge where beam loading is smaller. Depending on the electron bunch shape, beam loading can increase or decrease the energy spread, as different parts of the bunch experience different accelerating fields. In Fig. 4e, bunch lengths hardly change for different bump lengths of $L= 20, 70, 120$ $\upmu \hbox {m}$, with $\sigma _{FWHM}= 0.8$ $\upmu \hbox {m}$ and $\sigma _{rms}= 2.2$ $\upmu \hbox {m}$. Similarly, in Fig. 5e, the bunch length is constant for varying bump lengths, with $\sigma _{FWHM} =0.5$ $\upmu \hbox {m}$ and $\sigma _{rms}= 2.6$ $\upmu \hbox {m}$. Additionally, the properties of electron bunch slices for different bump lengths are similar in both Figs. 4 and 5. This implies that the bunch properties depend more on the down-ramp gradient of the bump than the length of the bump, as expected. The large difference in the bunch properties observed in Figs. 4 and 5 indicates that the injection point itself can be used as a parameter to modify the electron bunch properties. Tables 2 and 3 show electron bunch parameters corresponding to Figs. 4 and 5, respectively, where the slice values correspond to the position where the slice energy spread is smallest, $\Delta E_//\langle E_/ \rangle \approx 0.5\%$, in Table 2 corresponding to a peak current of 3–5 kA and normalised transverse slice emittance, $\varepsilon _/^\perp \approx 0.2 \pi$ mm mrad. Table 3 shows similar values but with higher current. To explore this further, a parameter scan is performed by varying the two bump parameters most easily manipulated in an experiment—the height and position of the bump (refer to Supplementary Fig. S1). The importance of laser evolution has been discussed in the context of electron self-injection in the LWFA^45,47,57,68^, but here we demonstrate that the laser evolution can be used as an extra control parameter for tuning the parameters of plasma-based electron accelerators.

**Table 2:** Bunch parameters for different bump lengths from Fig. 4.

Bump length, *L* [$\upmu$m]	Figure 4h pos. of $\Delta E_//\langle E_/ \rangle$ [$\upmu$m]	Figure 4h $\Delta E_//\langle E_/ \rangle$ [%]	Figure 4g *E* [MeV]	Figure 4g *dE*/*dz* [MeV/$\upmu$m]	Figure 4e *dQ*/*dz* [pC/$\upmu$m]	Figure 4e Current [kA]	Figure 4f $\varepsilon _/^\parallel$ [$\pi$ mm mrad]	Figure 4i $\varepsilon _/^\perp$ [$\pi$ mm mrad]
20	954.9	0.49	161	− 61.4	10.99	3.3	0.007	0.20
70	955.2	0.56	148	− 45.9	14.92	4.5	0.014	0.19
120	955.1	0.52	151	− 37.5	16.29	4.9	0.013	0.20

Slice values, $\Delta E_//\langle E_/ \rangle$, $\varepsilon _/^\parallel$ and $\varepsilon _/^\perp$, correspond to position where $\Delta E_//\langle E_/ \rangle$ is smallest.

**Table 3:** Bunch parameters for different bump lengths from Fig. 5.

Bump length [$\upmu$m]	Figure 5h pos. of $\Delta E_//\langle E_/ \rangle$ [$\upmu$m]	Figure 5h $\Delta E_//\langle E_/ \rangle$ [%]	Figure 5g *E* [MeV]	Figure 5g *dE*/*dz* [MeV/$\upmu$m]	Figure 5e *dQ*/*dz* [pC/$\upmu$m]	Figure 5e Current [kA]	Figure 5f $\varepsilon _/^\parallel$ [$\pi$ mm mrad]	Figure 5i $\varepsilon _/^\perp$ [$\pi$ mm mrad]
20	1453.1	0.91	117	− 12.4	23.42	7.0	0.024	0.34
	1454.5	0.89	114	8.4	86.66	26.0	0.023	2.15
70	1453.2	1.33	127	− 19.2	24.69	7.4	0.038	0.52
	1454.8	0.94	116	3.5	75.85	22.7	0.024	1.70
120	1453.3	1.75	128	− 30.4	22.77	6.8	0.049	0.43
	1454.8	0.85	116	6.2	74.91	22.5	0.022	2.30

Slice values, $\Delta E_//\langle E_/ \rangle$, $\varepsilon _/^\parallel$ and $\varepsilon _/^\perp$, correspond to position where $\Delta E_//\langle E_/ \rangle$ is smallest.

### Single down-ramp injection

Supplementary Fig. S1 shows analogous behaviour to that shown in Fig. 3. The highest charge is injected for the shortest ramp length, and as the gradient is reduced by increasing *R* the injected charge also reduces (c). The response of the bubble to the slower change in density causes a smaller change in the velocity of its back, which results in capture of less charge. The reduction in charge for longer ramps, in turn, leads to higher electron energy (d), in addition to lower slice spread (h) and slice emittance (f),(i). The decrease of electron energy with increasing charge for shorter ramps, as observed in Supplementary Fig. S1b,d, is explained by beam-loading reducing the accelerating field. The bunch lengths for different ramp lengths *R* in Supplementary Fig. S1e are: $\sigma _{FWHM}= 1.4 \ \upmu$m, $\sigma _{rms}= 3.2 \ \upmu$m ($R= 0 \ \upmu$m); $\sigma _{FWHM}= 0.8 \ \upmu$m, $\sigma _{rms}= 2.6 \ \upmu$m ($R= 25 \ \upmu$m); $\sigma _{FWHM}= 1.4 \ \upmu$m, $\sigma _{rms}= 2.0 \ \upmu$m ($R= 50 \ \upmu$m). This shows an increase in the electron bunch duration for shorter ramp lengths. Shorter ramps also tend to have higher slice energy spread (Supplementary Fig. S1h) because of increased beam loading. For ramp length $R>25$ $\upmu \hbox {m}$, the transverse emittance $\varepsilon _/^\perp$ remains less than $\sim 1 \ \pi$ mm mrad, shown in Supplementary Fig. S1f. For $R=0$, slice emittance is larger than the other two cases as electrons with larger transverse momentum are trapped due to the rapid change in bubble length similar to that in Fig. 3. Supplementary Table S1 shows the electron bunch parameters corresponding to Supplementary Fig. S1. the slice energy spread is $\Delta E_//\langle E_/ \rangle \approx 0.5\%$ and corresponding peak current $\approx$ 5 kA and slice emittance $\varepsilon _/^\perp \approx$ 0.1–0.4 $\pi$ mm mrad.

The evolution of the laser pulse in the plasma introduces a strong dependence of the accelerated electron bunch properties on the injection position. This is shown in the bump injection case by comparing Figs. 4 and 5, as well as explicitly in Supplementary Fig. S3. The impact of the down-ramp position on the evolution of the laser pulse is noteworthy. In the down-ramp case, the evolution is identical until the point of injection. However, at the injection point, the reduction in plasma density changes relativistic self-focusing and therefore the evolution of the laser pulse (as shown in Supplementary Fig. S4).

**Figure 6:**
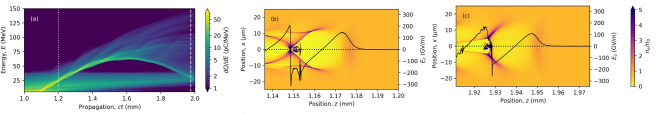
Energy spectrum evolution. (**a**) presents the time evolution of the electron energy spectrum, clearly showing an accelerated bunch that then undergoes a dephasing-like transition. (**b**) The density and on-axis accelerating field shortly after injection (indicated by the dotted line in (**a**)), while (**c**) shows that this bunch is then overtaken by the back of the bubble, with the majority of the bunch located in the decelerating phase ($E_z > 0$) of the wakefield (at the time indicated by the dashed line in (**a**)). The very front of the bunch continues to be accelerated, introducing the large energy spread at later times, observed in (**a**).

Figure 6a shows that the electron bunch injected at $z_i = 1$ mm undergoes a dephasing-like process, which leads to a reduction in energy and an increase in energy spread. An initially accelerated electron bunch is clearly identified (see Fig. 6b) before most of the charge begins to decelerate. Panel (c) shows that the bunch has not reached dephasing at the centre of the bubble, but rather the back of the bubble overtakes most of the bunch. This contraction is caused by the reduction in $a_0$ after the laser passes its focus, and in Fig. 6c we observe a smaller bubble with a weaker laser wakefield (compared to b). The wake also exhibits features of beam loading, as the high-charge bunch previously contained within the stronger laser wakefield now drives a comparable (if not stronger) wakefield that appears as a cone-like structure extending beyond the rear of the laser wake. The larger part of the bunch is now located in the decelerating field behind the bubble, while the front continues to be accelerated. This introduces a large energy spread as observed at later times in (a) and Fig. 2d.

In Supplementary Fig. S2, we show the evolution of electron bunch properties with respect to the injection position. The values in the shaded region ($z_i > 1$ mm) can not be determined precisely as most of the charge is decelerated below the energy selection threshold 20 MeV (as in Fig. 2d). Supplementary Figure S2a shows the total injected charge. For a given single down-ramp, the change of $a_0$ with the injection position $z_i$ plays a key role in the injection: higher values of $a_0$ yield a higher injected charge. The increase in injected charge can be explained by several effects arising from the change in the laser amplitude: The ponderomotive force of the laser on the electrons increases with $a_0$, which enlarges the bubble^69^ and displaced electron volume, as well as imparting larger momentum to electrons. The larger bubble leads to higher field strengths and maximum energy of injected electrons. However, beam loading due to the increase in charge typically lowers the mean energy, as shown in Supplementary Fig. S2b,e, and, by deforming the electric field distribution, increases the energy spread, as shown in Supplementary Fig. S2c. Supplementary Table S2 shows the minimum slice parameters for Supplementary Fig. S2, where the slice energy spread of $\Delta E_//\langle E_/ \rangle \approx 0.5\%$ at 165 MeV bunch can be achieved with a peak current of $\approx$ 6 kA and a slice transverse emittance, $\varepsilon _/^\perp \approx 0.5\,\pi$ mm mrad. It is worth noting that the bunch duration ($\sigma _{rms}= 3.0$ $\upmu \hbox {m}$) remains almost constant with increasing injected charge, thus we are able to tune the bunch charge by varying the injection position $z_i$, while maintaining its duration. As expected, the slice energy spread in Supplementary Fig. S2h and the slice emittance in (f) and (i) are both reduced for lower injected charge. To extend the investigation on bunch properties, a parameter scan is performed by varying the down-ramp parameters (Supplementary Fig. S4)

The above discussion indicates that the injected charge can be indirectly controlled by tuning the laser $a_0$ through choice of the position of the down-ramp. However, if a density down-ramp is used to trigger injection after the laser focus, where the amplitude is dropping, then the bubble size reduces and the electron bunch can experience a dephasing-like process at the back of the bubble. The advantage is that this places the front of the bunch in a very high field to attain a much higher maximum energy, but with the consequence that the average energy and energy spread of the whole bunch are much worse. In this regard, a more stable or guided laser evolution might be beneficial for acceleration to high energy over long lengths^70^. When choosing the laser parameters, it is important to ensure that the laser does not cause self-injection before the stimulated injection (beam loading can then help suppress further dark current), which places restrictions on the choice of laser and plasma parameters.

Overall, we show the possibility of tailoring the parameters of the electron bunches produced in LWFAs by tuning the down-ramp height as well as the injection point. We demonstrate that the amount of charge, the bunch length, and the emittance can be controlled by changing the height and position of the down-ramp, which is experimentally easier than changing its shape. From this parameter scan, we can determine optimum parameters for various applications of the LWFA.

## Conclusion

We have presented a study of a method of producing high-quality electron bunches using self-injection at a density bump and a single down-ramp. We show that the electron bunch properties can be tuned by varying the plasma density bump/ramp-length, position, and height. For square bump ($R=0$), we observe increased electron injection and better quality bunches can be obtained by tuning the bump height and position. We have shown how the injection position determines the electron bunch charge and depends on the evolution of the laser pulse $a_0$. To ensure identical laser evolution up to the point of injection, we have performed simulations for single down-ramp injection. Simulations for different density bump lengths suggest that the electron bunch properties are predominantly influenced by the position of the down-ramp gradient rather than by the bump length. Parameter scans are plotted for large data sets and thus precise parameters can be obtained according to the requirements. In the parameter scan for height $\alpha$ and injection position $z_i$, regions of lower average slice emittance and slice energy spread give preferred parameters for applications. With this analysis we show that an efficient strategy can be found for controlling the electron bunch parameters—charge, energy, emittance and energy spread. We show that the slice energy spread can be $\Delta E_//\langle E_/ \rangle < 0.5\%$ at 165 MeV, and with a peak current of $\approx$ 6 kA and slice emittance as small as $\varepsilon _/^\perp \approx 0.05 \,\pi$ mm mrad. If the emittance can be preserved during subsequent acceleration a much smaller energy spread can be achieved. This could make LWFAs suitable for driving XUV and X-ray FELs using a simple and controllable self-injection scheme.

We anticipate that taking into account laser pulse evolution in optimization and control of self-injection to produce high-quality electron bunches may be crucial for developing compact coherent light sources. Bump and down-ramp parameters enable good control over electron bunch properties. The determination of precise parameter values will require further studies to address the varying requirements of different applications and accommodate available experimental conditions.

## Methods

We have performed a series of simulations using the quasi-3D PIC code FBPIC^71^, which uses cylindrical geometry and azimuthal modal decomposition to represent the 3D domain. In larger simulation domains and for longer propagation, PIC algorithms can be very computationally demanding and are subject to substantial numerical artifacts. Spectral field solvers, as used by FBPIC, exhibit no spurious numerical dispersion or numerical Cherenkov radiation, and can significantly reduce other forms of numerical noise that arise using finite difference schemes. The simulations included three modes ($m = 0,1,2$), and a $z \times r$ = 63.76 $\upmu \hbox {m}$ $\times$ 80 $\upmu \hbox {m}$ moving window travelling at the speed of light (in vacuum, *c*), with grid cell size $\Delta z = \Delta r = 40$ nm and (3,2,12) macro-particles per cell in (*z*,*r*,$\theta$). The plasma target has a plateau electron density $n_0 = 3\times 10^{18}$ cm$^{-3}$ and 100 $\upmu \hbox {m}$ sinusoidal entrance and exit ramps. A linearly-polarized Gaussian laser pulse with wavelength $\lambda = 800$ nm, normalized amplitude $a_0 = 2.5$, waist $w_0 = 15$ $\upmu \hbox {m}$, and full-width at half-maximum pulse duration 33 fs, propagates along the *z*-axis and is focussed at the bottom of the up-ramp to the plasma plateau. The laser parameters are chosen to minimize self-injection (total charge $Q < 0.1$ pC) during the interaction with the flat-top plasma (low dark current) despite the laser self-focusing to around $a_0 \sim 4$.

### Supplementary Information

Supplementary Information.

## Data Availability

Data associated with research published in this paper is available at: https://doi.org/10.15129/7b3da154-f717-4b15-8118-abe3828c7c56.
