# Supplementary material for: The effect of laser pulse evolution on down-ramp injection in laser wakefield accelerators
Source: Sci Rep. 2024 Aug 19;14:19127. doi: 10.1038/s41598-024-69049-4 (PMC11330977; doi:10.1038/s41598-024-69049-4)
Supplement: Supplementary file 1 — Supplementary Information. [file 41598_2024_69049_MOESM1_ESM.pdf]

The effect of laser pulse evolution on down-ramp injection in laser wakefield accelerators

Arohi Jain[1,+], Samuel R. Yoffe[2,+], Bernhard Ersfeld[2], George K. Holt[2], Devki Nandan Gupta[1,†], and Dino A. Jaroszynski[2,*]

[1]Department of Physics and Astrophysics, University of Delhi, Delhi 110 007, India
[2]Department of Physics, SUPA & University of Strathclyde, Glasgow G4 0NG, United Kingdom
[*]Corresponding author: d.a.jaroszynski@strath.ac.uk
[†]Corresponding author: dngupta@physics.du.ac.in

Supplementary Information and Figures

Single down ramp injection

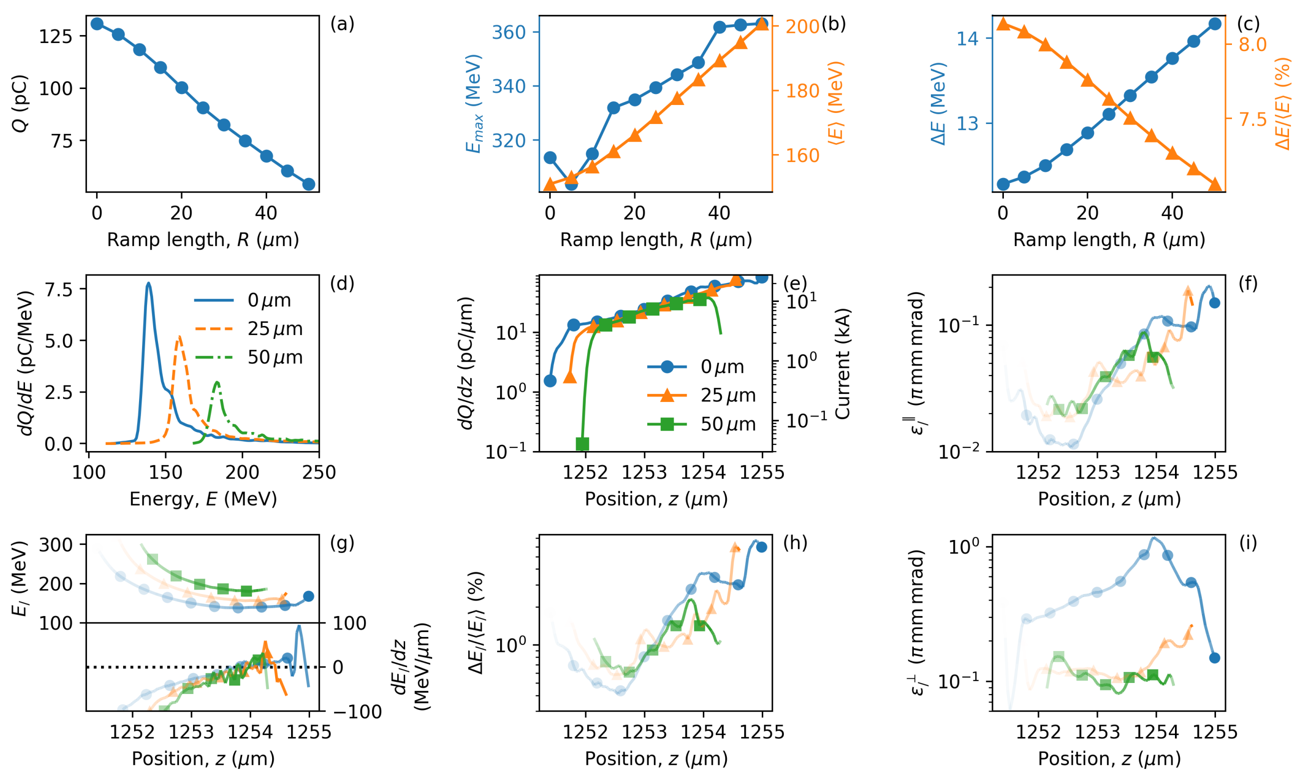

Figure S1. Variation in electron bunch properties with ramp lengths. Electron bunch properties for a single down ramp at $z_i = 300\,\mu\text{m}$ with $\alpha = 0.3$. Panels (a)-(c) show the variation of the injected charge, energy, and energy spread, respectively. Data points indicated by circles correspond to the left axis, and triangles to the right axis. Panels (d) and (e) show the energy spectra and charge distribution, respectively, for three ramp lengths, $R = 0, 25, 50\,\mu\text{m}$. Properties of electrons contained in a narrow slice are presented in panels (f)-(i), where opacity is used to indicate charge density and the same legend as panel (e) is used to identify lines. Properties are calculated at $ct = 1.3\,\text{mm}$ (1 mm after injection).

Table S1. Bunch parameters for different bump lengths from Fig. S1. Slice values, $\Delta E_/ / \langle E_/ \rangle$, $\varepsilon_/^{\parallel}$ and $\varepsilon_/^{\perp}$, correspond to position where $\Delta E_/ / \langle E_/ \rangle$ is smallest.

**Ramp length [μm]**	**Fig. S1(h) pos. of $\Delta E_/ / \langle E_/ \rangle$ [μm]**	**Fig. S1(h) $\Delta E_/ / \langle E_/ \rangle$ [%]**	**Fig. S1(g) E [MeV]**	**Fig. S1(g) dE/dz [MeV/μm]**	**Fig. S1(e) dQ/dz [pC/μm]**	**Fig. S1(e) Current [kA]**	**Fig. S1(f) $\varepsilon_/^{\parallel}$ [π mm mrad]**	**Fig. S1(i) $\varepsilon_/^{\perp}$ [π mm mrad]**
**0**	1252.6	0.41	166	-38.8	18.48	5.5	0.011	0.38
**25**	1252.5	0.58	203	-68.0	15.32	4.6	0.019	0.12
**50**	1252.7	0.58	223	-79.6	17.67	5.3	0.020	0.11

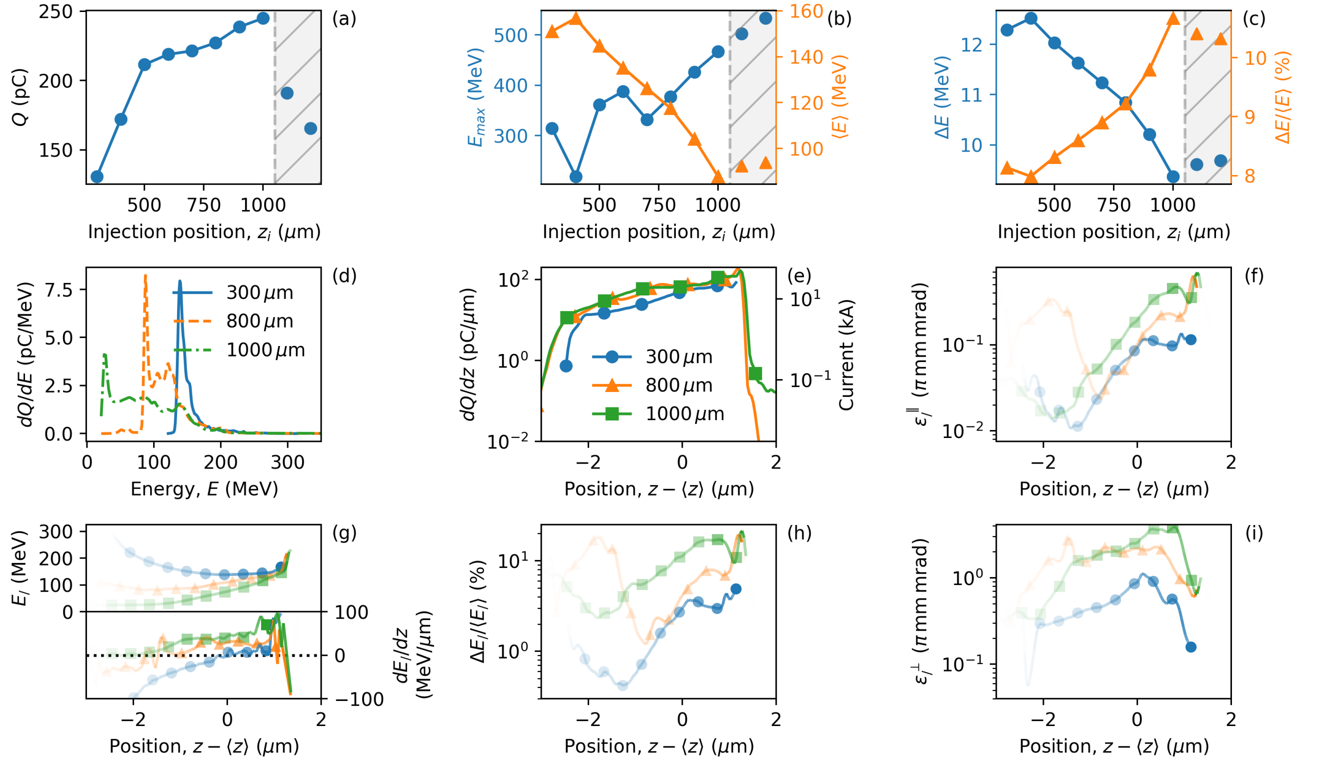

Figure S2. Variation in electron bunch properties with down-ramp position. Electron bunch properties for injection by a single down ramp at z_i with $\alpha = 0.3$. (a)-(c) show the variation of the injected charge, energy, and energy spread, respectively. Data points indicated by circles correspond to the left axis, and triangles to the right axis. Data points for $z_i > 1$ mm (grey shaded region) are shown for completeness, but much of the charge has been decelerated to below the particle selection energy threshold (20 MeV) preventing accurate determination of the bunch properties. (d) and (e) show the energy spectra and charge distribution, respectively, for three example injection positions, $z_i = 0.3, 0.8, 1$ mm. Properties for electrons contained in a narrow slice are presented in (f)-(i), where opacity is used to indicate charge density and the same legend as (e) is used to identify lines. Properties are calculated at $ct = 1.3$, 1.8 and 2 mm (1 mm after injection).

Table S2. Bunch parameters for different downramp positions from Fig. S2. Slice values, $\Delta E_{/}/\langle E_{/}\rangle$, $\varepsilon_{/}^{\parallel}$ and $\varepsilon_{/}^{\perp}$, correspond to position where $\Delta E_{/}/\langle E_{/}\rangle$ is smallest.

Injection pos., zi [μm]	Fig. S2(h) pos. of $\Delta E_{/}/\langle E_{/}\rangle$ [μm]	Fig. S2(h) $\Delta E_{/}/\langle E_{/}\rangle$ [%]	Fig. S2(g) E [MeV]	Fig. S2(g) dE/dz [MeV/μm]	Fig. S2(e) dQ/dz [pC/μm]	Fig. S2(e) Current [kA]	Fig. S2(f) $\varepsilon_{/}^{\parallel}$ [π mm mrad]	Fig. S2(i) $\varepsilon_{/}^{\perp}$ [π mm mrad]
**300**	1252.6	0.42	165	-39.3	18.63	5.6	0.011	0.39
	1254.6	2.97	145	-6.4	71.48	21.4	0.097	0.57
**800**	1752.0	1.20	88	12.5	54.10	16.2	0.024	2.12
	1752.4	2.12	100	40.1	75.65	22.7	0.046	1.93
	1753.6	6.80	135	57.7	96.02	28.8	0.207	1.03
**1100**	1950.4	2.31	26	3.8	24.92	7.5	0.014	0.61
	1953.3	9.27	143	55.5	120.28	36.1	0.299	1.10

Parameter Scans

Figure S3 shows the (a) total charge, (c) average energy, (d) energy spread, (e) average slice energy spread, and (b) sum of the squares of average longitudinal and transverse slice emittance of the injected electron bunch in the PIC simulations. The injected charge is higher in the region where $\alpha > 0.3$ and $z_i > 0.8$ mm. The important point to note from this parameter scan is that the injected charge can be manipulated by choosing the position of the bump, corresponding to different times of laser

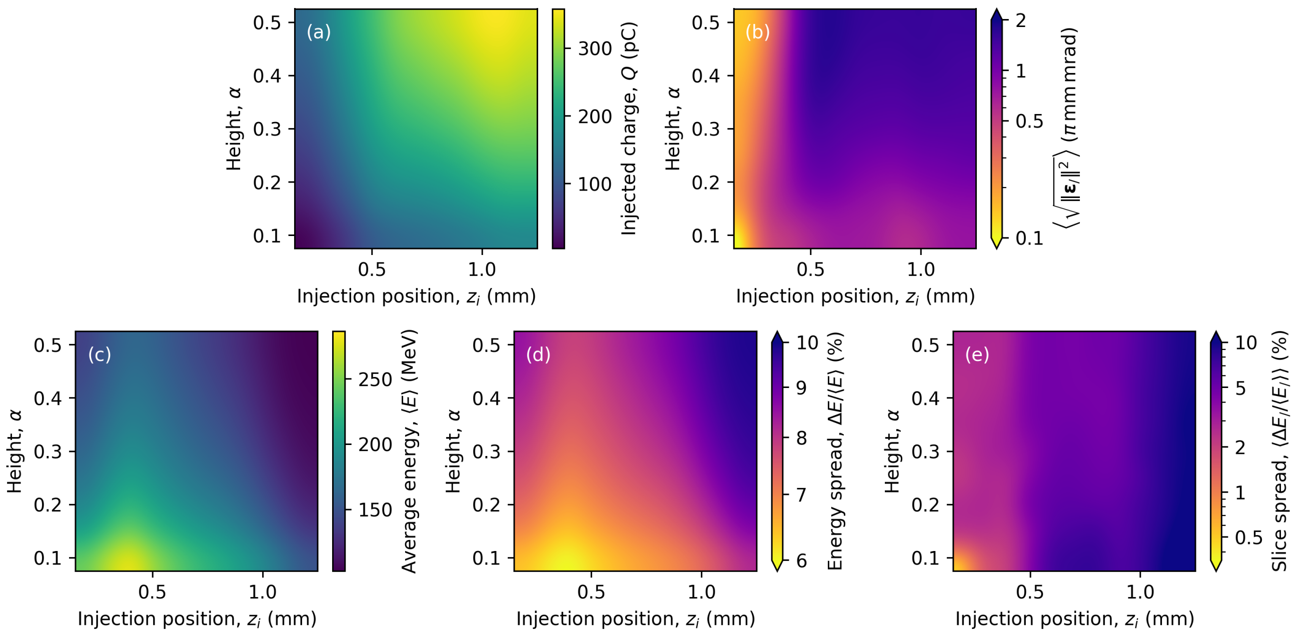

Figure S3. **Parameter scans.** Scan for bump height α and injection position z_i. Panels (a) and (c) show the injected charge and average energy, respectively, while (d) and (e) show the total energy spread and average slice energy spread. Panel (b) gives an indication of bunch quality by taking the sum of the squares of the average slice emittance components, with a lower value being better for FEL applications. Properties are calculated at $ct = z_i + 1$ mm (1 mm after injection) considering electrons injected into the first bubble only. Note that interpolation has been used to produce smooth colour gradients.

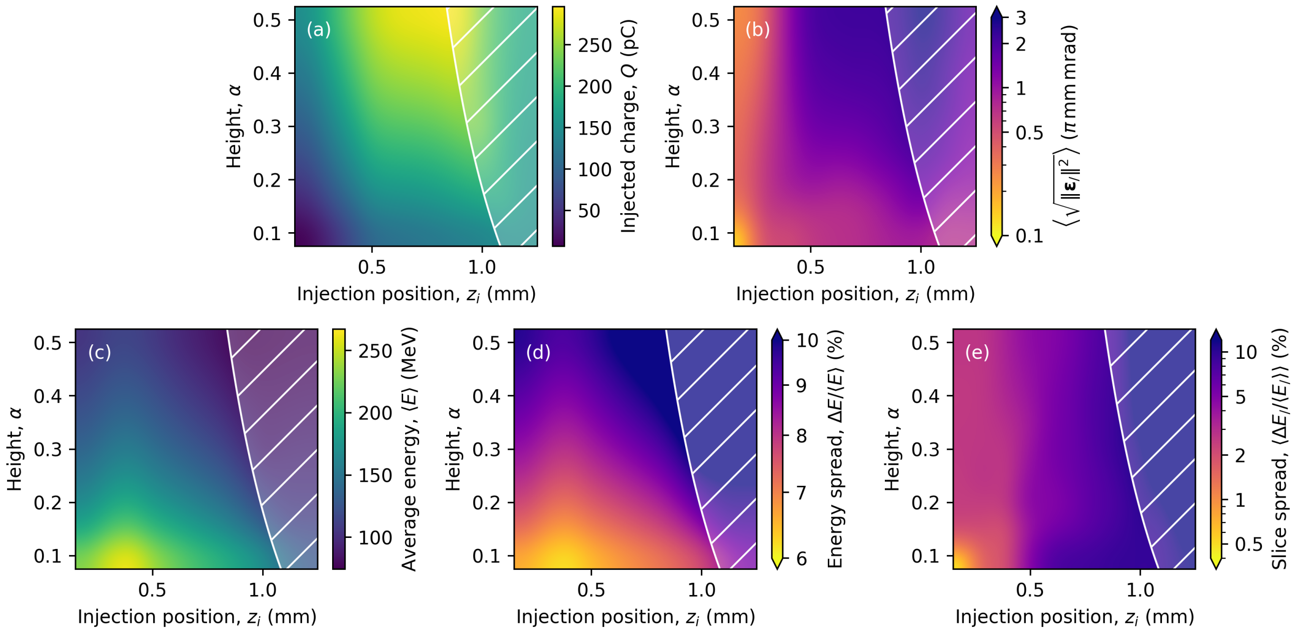

Figure S4. Parameter scans. Scan for down-ramp height α and injection position z_i. (a) and (c) show the injected charge and average energy, respectively, while (d) and (e) show the total energy spread and average slice energy spread. (b) gives an indication of bunch quality by taking the sum of the squares of the average slice emittance components, with a lower value being better for FEL applications. Properties are calculated at $ct = z_i + 1$ mm (1 mm after injection). The hatched region indicates where the electron bunch could not be accurately determined. Note that interpolation has been used to produce smooth colour gradients.

evolution. The injected charge depends on the laser normalised vector potential, a_0, because it directly determines the wake phase velocity in the non-linear regime[1], but increasing a_0 also increases the electron momentum, allowing more electrons to satisfy the conditions for injection. Furthermore, in the non-linear regime the wakefield bubble size increases with a_0, increasing the injected bunch charge[2]. The total amount of injected charge increases with α because both the number of electrons available at the injection point and the rate of bubble expansion (due to the density gradient) increase with bump height. From the results of the parameter scan, we observe that better quality electron bunches are obtained for early injection, while the laser amplitude is increasing, in this case around $z_i < 0.4$ mm. After this point, more electrons are injected at the cost of lower bunch quality. However, different applications of LWFAs require particular sets of electron bunch parameters. For example, to use the electron bunch as a driver of a compact FEL, one would typically prefer short-duration, low-emittance beams observed for $z_i < 0.4$ mm, which demonstrate high current. On the other hand, for production of betatron X-rays (synchrotron-like during the acceleration process), increasing the bump height and injecting near the focal plane are preferred as it results in high charge and large transverse emittance (*i.e.* larger betatron oscillation amplitude).

Once again, a parameter scan is performed for the down-ramp height and position, and the electron bunch properties are shown in Fig. S4. In panel (a), the amount of trapped charge is shown to increase for higher density transitions due to rapid expansion of the bubble, in addition to the availability of more of electrons. As discussed previously, the injected charge increases with a_0, thus higher charge is injected near the focal position ($z_i \sim 0.8$ mm). To maximize the amount of charge in the electron bunch, a sharp density transition and an injection position near the focal position of the laser pulse should be chosen. The mean energy is higher in the lower charge region, as shown in Fig. S4(c), because beam loading reduces the accelerating field for higher bunch charge. Thus, to achieve higher mean energy $\alpha < 0.2$ and $z_i < 0.6$ mm should be chosen. In Fig. S4(d), a total energy spread $\sim 6\%$ with $\langle E\rangle \sim 250$ MeV is reported for an injection position less than 0.6 mm and ramp height, α, around 0.1. In addition, the average slice energy spread shown in Fig. S4(e) is less than 1% for $\alpha < 0.2$ and $z_i < 0.4$ mm. Interestingly, we observe regions of lower emittance for both lower and higher charge values, if the injection point is chosen around 0.2 mm.

References

1. Tooley, M. P. *et al.* Towards attosecond high-energy electron bunches: Controlling self-injection in laser-wakefield accelerators through plasma-density modulation. *Phys. Rev. Lett.* **119**, 044801, DOI: 10.1103/PhysRevLett.119.044801 (2017).
2. Benedetti, C., Schroeder, C. B., Esarey, E., Rossi, F. & Leemans, W. P. Numerical investigation of electron self-injection in the nonlinear bubble regime. *Phys. Plasmas* **20**, 103108, DOI: 10.1063/1.4824811 (2013).
